# Molecular Cloning and Characterization of Porcine Na^+^/K^+^-ATPase Isoforms α1, α2, α3 and the ATP1A3 Promoter

**DOI:** 10.1371/journal.pone.0079127

**Published:** 2013-11-13

**Authors:** Carina Henriksen, Kasper Kjaer-Sorensen, Anja Pernille Einholm, Lone Bruhn Madsen, Jamal Momeni, Christian Bendixen, Claus Oxvig, Bente Vilsen, Knud Larsen

**Affiliations:** 1 Department of Molecular Biology and Genetics, Aarhus University, Tjele, Denmark; 2 Department of Biomedicine, Aarhus University, Aarhus C, Denmark; 3 Department of Molecular Biology and Genetics, Aarhus University, Aarhus C, Denmark; Universidade Federal do Rio de Janeiro, Brazil

## Abstract

Na^+^/K^+^-ATPase maintains electrochemical gradients of Na^+^ and K^+^ essential for a variety of cellular functions including neuronal activity. The α-subunit of the Na^+^/K^+^-ATPase exists in four different isoforms (α1–α4) encoded by different genes. With a view to future use of pig as an animal model in studies of human diseases caused by Na^+^/K^+^-ATPase mutations, we have determined the porcine coding sequences of the α1–α3 genes, *ATP1A1*, *ATP1A2*, and *ATP1A3*, their chromosomal localization, and expression patterns. Our *ATP1A1* sequence accords with the sequences from several species at five positions where the amino acid residue of the previously published porcine *ATP1A1* sequence differs. These corrections include replacement of glutamine 841 with arginine. Analysis of the functional consequences of substitution of the arginine revealed its importance for Na^+^ binding, which can be explained by interaction of the arginine with the C-terminus, stabilizing one of the Na^+^ sites. Quantitative real-time PCR expression analyses of porcine *ATP1A1*, *ATP1A2*, and *ATP1A3* mRNA showed that all three transcripts are expressed in the embryonic brain as early as 60 days of gestation. Expression of α3 is confined to neuronal tissue. Generally, the expression patterns of *ATP1A1*, *ATP1A2*, and *ATP1A3* transcripts were found similar to their human counterparts, except for lack of α3 expression in porcine heart. These expression patterns were confirmed at the protein level. We also report the sequence of the porcine *ATP1A3* promoter, which was found to be closely homologous to its human counterpart. The function and specificity of the porcine *ATP1A3* promoter was analyzed in transgenic zebrafish, demonstrating that it is active and drives expression in embryonic brain and spinal cord. The results of the present study provide a sound basis for employing the *ATP1A3* promoter in attempts to generate transgenic porcine models of neurological diseases caused by *ATP1A3* mutations.

## Introduction

The Na^+^/K^+^-ATPase (sodium/potassium pump), first described in 1957 [1], is a membrane bound ion pump belonging to the family of P-type ATPases. Several members of this family catalyze active transport of cations across the cell membrane and function in maintaining the ionic gradients through hydrolysis of ATP. The Na^+^/K^+^-ATPase pumps sodium ions out of the cell and potassium ions into the cell with a stoichiometry of 3Na^+^ for 2K^+^ [2], [3]. It is a hetero-oligomer composed of α- and β-subunits [4] as well as in many tissues of a regulatory subunit belonging to the FXYD protein family. The α-subunit consists of ten transmembrane helices (M1–M10), harboring the binding sites for Na^+^ and K^+^, and three cytoplasmic domains, the actuator (A), the nucleotide-binding (N), and the phosphorylation (P) domain, which are involved in ATP hydrolysis [5], [6]. The Na^+^/K^+^-ATPase α-subunit exists in four different isoforms, α1, α2, α3, and α4, encoded by four different genes, *ATP1A1*, *ATP1A2*, *ATP1A3*, and *ATP1A4*, respectively [7], [8]. The four α-isoforms have a high degree of amino acid identity, although their expression pattern within mammalian tissues and cell types vary [9]. The α1-isoform seems to be uniformly expressed in all cells, while the α2-isoform is reported to be predominantly expressed in astrocytes (glial cells) and muscle, and the α3-isoform is primarily found expressed in neurons [10]–[15], although expression in human heart is reported [16], [17], [18]. The α4-isoform is only found in sperm cells and is essential for male fertility [7], [19].

Deficient function of the α2- or α3-isoform of the Na^+^/K^+^-ATPase, caused by mutations in the genes *ATP1A2* or *ATP1A3*, is associated with the neurological diseases hemiplegic migraine (HM) and rapid-onset dystonia parkinsonism (RDP), respectively [20]–[22]. RDP, first described in 1993 [23], is a movement disorder characterized by sudden onset of dystonia often with signs of parkinsonism. Recently, mutations in the *ATP1A3* gene were found associated with another neurological disease, alternating hemiplegia of childhood (AHC), as well [24], [25]. The pathophysiological mechanisms underlying these disorders are poorly understood.

Detailed studies of human pathophysiology are often hampered or prevented because of ethic considerations and rules. Pig is the nearest related accessible non-primate animal, and during recent years focus on porcine models for studying human diseases has gradually increased [26], [27]. The anatomical, physiological, genetic, and biochemical resemblance between man and pig is very close. Furthermore, the development and the topical, histological, and vascular anatomy of the pig brain makes it very useful as a model for investigation of neurological diseases in humans [28]. In order to obtain information pertinent to human and to investigate the potential of developing a porcine model for RDP/AHC, we have cloned the porcine *ATP1A1*, *ATP1A2*, and *ATP1A3* coding sequences found expressed in brain, and the promoter region of *ATP1A3*. We report here structural and functional investigations in relation to these genes and the *ATP1A3* promoter, as well as the spatial expression pattern in adult pigs and in embryos of different development stages. The function of the *ATP1A3* promoter was tested in transgenic zebrafish. The present results form the basis for employing the *ATP1A3* promoter in attempts to generate transgenic pigs overexpressing mutated porcine *ATP1A3* genes containing RDP/AHC mutations. During the course of these experiments we furthermore found several differences relative to the previously published porcine *ATP1A1* coding sequence. An arginine interacting with the C-terminus was previously assigned as a glutamine, and we found it important for Na^+^ binding.

## Results and Discussion

### Identification of the porcine *ATP1A1* coding sequence

**Figure 1:**
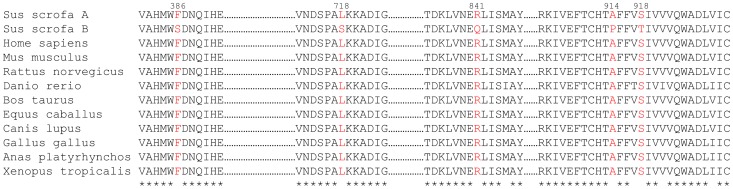
Comparison of the porcine Na^+^/K^+^-ATPase α1 amino acid sequence with previously published α1 sequences from pig and other species. Pig (Sus scrofa A, GQ340774 and Sus scrofa B, NM214249), human (Homo sapiens, NM000701), mouse (Mus musculus, NM144900), rat (Rattus norvegicus, NM012504), zebrafish (Danio rerio, NM131686), cattle (Bos taurus, NM001076798), horse (Equus caballus, NM001114532), dog (Canis lupus, NM001003306), chicken (Gallus gallus, NM205521), duck (Anas platyrhynchos, EU004277), and frog (Xenopus tropicalis, NM204076). The amino acids indicated in red show the five positions where our porcine sequence (GQ340774) differs from the previously described porcine Na^+^/K^+^-ATPase α1-sequence (NM214249). The numbering of the residues refers to the porcine sequence after posttranslational cleavage of the first five amino acid residues. * indicates identity in all the species.

The pig *ATP1A1* coding sequence has previously been reported (GenBank ID: NM_214249). Notably, the previously published porcine sequence (Sus scrofa B in Fig. 1) differs from that of several other species, including human, at certain amino acid positions, where there is a high degree of conservation across most species (Fig. 1). To clarify this issue, we re-cloned the porcine *ATP1A1* coding sequence. When comparing the new sequence of Na^+^/K^+^-ATPase α1-polypeptide (Sus scrofa A, GenBank ID: GQ340774) with the previously described porcine α1-sequence (Sus scrofa B), we observed five amino acid differences (Fig. 1). As in other species, a leucine was found in our porcine α1-sequence (Sus scrofa A) in place of the previously reported serine at position 718 in the regulatory K^+^ site of the P-domain [29]. Furthermore, a phenylalanine was found in place of a serine at position 386 in the N-domain, an arginine in place of a glutamine at position 841 at the cytoplasmic end of transmembrane segment M7, an alanine in place of a proline at position 914, and a serine in place of a threonine at position 918, the latter two being located in transmembrane segment M8. These changes (indicated in red) accord with the residues found at the corresponding positions in several other species (Fig. 1).

### The arginine at position 841 is functionally important

**Figure 2:**
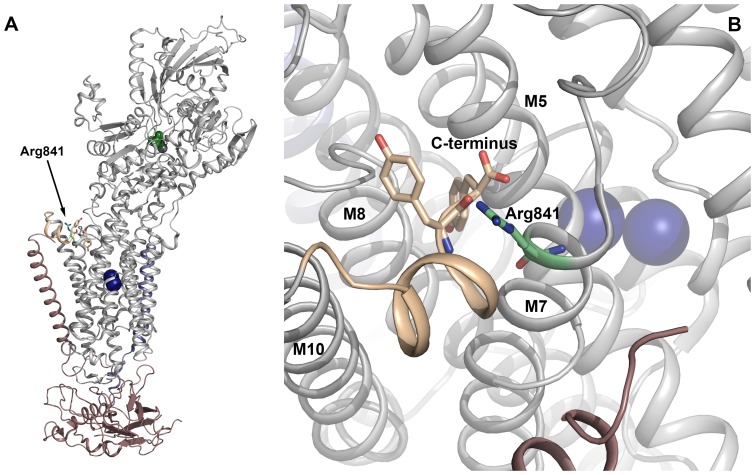
Structural relation of Arg841 to the C-terminus. The high resolution crystal structure of the K^+^-bound E2 form of shark Na^+^/K^+^-ATPase [6] is shown (a structure of the Na^+^ bound E1 form has not been published). The shark Na^+^/K^+^-ATPase has an arginine at the position corresponding to Arg841 in the porcine α1-sequence presented here, and the relation of the arginine to the C-terminus can therefore be visualized using the structure of the shark enzyme. **A.** Overview of the crystal structure (cytoplasmic side up). The α-subunit is shown in *grey*, except the 11 most C-terminal residues, which are colored *wheat*. The β-subunit is *violet*, the FXYD protein is *blue*, and the two bound K^+^ ions are depicted as *dark blue spheres*. The two C-terminal tyrosines and the arginine homologous to Arg841 in pig are shown as *wheat* and *green* sticks, respectively. MgF_4_^2−^ bound as phosphate analog in the cytoplasmic P-domain is colored *green*. **B.** Close up of the arginine homologous to Arg841 in pig showing its closeness to the C-terminal carboxyl group as well as the main-chain carbonyl oxygen of the penultimate tyrosine. These interactions would not be possible with the glutamine erroneously assigned to position 841 in the previously published porcine α1-sequence.

**Figure 3:**
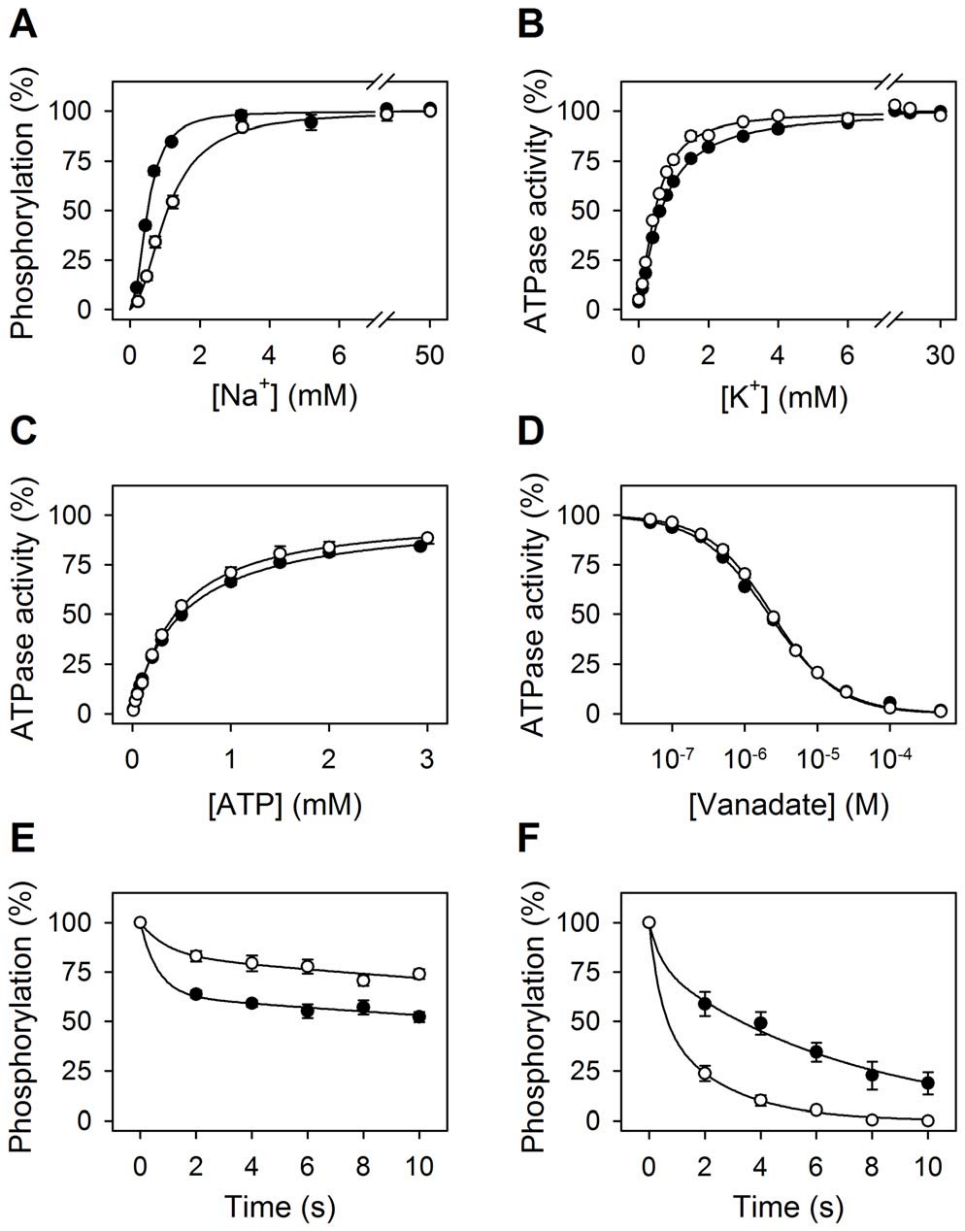
Functional importance of Arg841. The rat α1 Na^+^/K^+^-ATPase Arg843 homologous to pig Arg841 was replaced by alanine (“mutant”) and the functional consequences analyzed (see also Methods). Wild type, *closed circles*; mutant, *open circles*. The standard errors are indicated as error bars (seen only when larger than the size of the symbols). **A.** Na^+^ dependence of phosphorylation. Phosphorylation was carried out for 10 s at 0°C in the presence of 2 µM [γ-^32^P]ATP in P-medium with oligomycin and the indicated concentrations of Na^+^. Each *line* shows the best fit of the Hill equation, giving *K*_0.5_(Na^+^) values of 0.50±0.01 mM for wild type and 1.07±0.04 mM for the mutant. **B.** K^+^ dependence of Na^+^/K^+^-ATPase activity. The ATPase activity was measured at 37°C in A-medium with 40 mM Na^+^, 3 mM ATP, and the indicated concentrations of K^+^. Each *line* shows the best fit of the Hill equation, giving *K*_0.5_(K^+^) values of 0.67±0.01 mM for wild type and 0.50±0.02 mM for the mutant. **C.** ATP dependence of Na^+^/K^+^-ATPase activity. The ATPase activity was measured at 37°C in A-medium with 130 mM Na^+^, 20 mM K^+^, and the indicated concentrations of ATP. Each *line* shows the best fit of the Hill equation, giving *K*_0.5_(ATP) values of 0.50±0.03 mM for wild type and 0.43±0.04 mM for the mutant. **D.** Vanadate dependence of Na^+^/K^+^-ATPase activity. The ATPase activity was measured at 37°C in A-medium with 130 mM Na^+^, 20 mM K^+^, 3 mM ATP, and the indicated concentrations of vanadate. Each *line* shows the best fit of the Hill equation for inhibition, giving *K*_0.5_(vanadate) values of 2.2±0.1 µM for wild type and 2.4±0.1 µM for the mutant. **E.** Distribution of phosphoenzyme intermediates between E1P and E2P. Phosphorylation was carried out for 10 s at 0°C in the presence of 2 µM [γ-^32^P]ATP in P-medium with 20 mM Na^+^. Dephosphorylation was initiated by addition of 1 mM non-radioactive ATP and 2.5 mM ADP and terminated by acid quenching at the indicated times. Each *line* shows the best fit of a bi-exponential decay function giving amplitudes (corresponding to E2P) for the slow phase of 63±4% for wild type and 84±8% for the mutant. **F.** Rate of E1P→E2P interconversion. Phosphorylation was carried out for 15 s at 0°C in the presence of 2 µM [γ-^32^P]ATP in P-medium with 600 mM Na^+^. Dephosphorylation was initiated by addition of a chase solution producing final concentrations of 600 mM Na^+^, 20 mM K^+^, and 1 mM non-radioactive ATP in addition to the components in the P-medium, and terminated by acid quenching at the indicated times. Each *line* shows the best fit by a bi-exponential decay function giving rate constants for the slow phase (corresponding to the E1P→E2P interconversion) of 0.14±0.05 s^−1^ for wild type and 0.43±0.18 s^−1^ for the mutant.

It is of note that the structure of the pig Na^+^/K^+^-ATPase α1 determined by X-ray crystallography was modeled on the basis of the previously published porcine sequence, where a glutamine is present at position 841 [5], whereas the crystal structure of shark Na^+^/K^+^-ATPase, like the present porcine sequence, has an arginine at this position [6]. We speculated that this substitution of glutamine for arginine at position 841 might be of functional relevance, because the positively charged arginine side chain seems to interact electrostatically with the negatively charged C-terminal part of the Na^+^/K^+^-ATPase α1-polypeptide (Fig. 2). The Na^+^/K^+^-ATPase C-terminus has been shown to be important for Na^+^ binding, as it docks in between transmembrane helices M5, M7, M8, and M10, probably stabilizing one of the three Na^+^ binding sites supposed to be located between M5 and M8 [5], [30]–[32]. The functional importance of the putative interaction of the C-terminus with the arginine was studied by characterizing a mutant in which the arginine was replaced by alanine (Fig. 3). The mutation homologous to pig R841A was introduced into the ouabain resistant rat α1-isoform (R843A), and wild type and mutant were overexpressed under ouabain-selective pressure using the mammalian COS cell system [33]. Functional characterization was performed on isolated plasma membrane fractions. Because Na^+^ binding at the sites facing the cytoplasm is required to trigger phosphorylation from ATP (cf. Scheme S1), it is possible to analyze the Na^+^ affinity of these sites by measuring the phosphorylation from [γ-^32^P]ATP at various Na^+^ concentrations. As seen in Fig. 3A, the mutant displayed a 2-fold reduction in affinity for Na^+^ relative to the wild-type enzyme. Binding of K^+^ to sites on E2P facing the extracellular side activates dephosphorylation and thereby ATP hydrolysis (Scheme S1). Fig. 3B shows that the K^+^ dependence of the Na^+^/K^+^-ATPase activity of the mutant is wild type-like, thus indicating a normal affinity for K^+^. Because a shift of the equilibrium between the two conformational states of the unphosphorylated enzyme, E1 and E2, in favor of E2 would indirectly reduce the apparent affinity for Na^+^, assays were performed to characterize the E1–E2 equilibrium. If the mutation caused a shift of the equilibrium toward the E2-form, the apparent affinity for ATP in the activation of Na^+^/K^+^-ATPase activity should decrease, because ATP binds with high affinity to E1 and with low affinity to E2 [2], [31], [32], [34]. Furthermore, because vanadate is known to react with E2, and not with E1 [35], an equilibrium shift toward E2 should result in an increased affinity for vanadate in inhibition of Na^+^/K^+^-ATPase activity. As seen in Fig. 3C and Fig. 3D, respectively, the mutant displayed wild type-like affinity for both ATP and vanadate, indicating that the 2-fold reduction of Na^+^ affinity observed for the mutant represents a true decrease of the intrinsic affinity of the E1-form for Na^+^ and not an indirect effect of a shift of the E1–E2 equilibrium. This result suggests that the arginine is involved in the binding of Na^+^ to the cytoplasmically facing sites of the Na^+^/K^+^-ATPase, probably owing to its interaction with the C-terminus, helping to position the C-terminus correctly in relation to the Na^+^ binding site.

To assess the distribution of the phosphoenzyme between the two forms E1P (ADP sensitive, because it reacts backwards with ADP forming ATP) and E2P (ADP insensitive, but K^+^ sensitive, cf. Scheme S1), ADP was added to the phosphorylated enzyme, and the dephosphorylation was followed (Fig. 3E). Two decay phases could be distinguished, a rapid phase corresponding to the reaction of E1P with ADP and a slow phase corresponding to decay of E2P, the latter occurring at a low rate in the absence of K^+^. By fitting a bi-exponential decay function to the data in Fig. 3E, it can be estimated that the fraction of phosphoenzyme present as E2P at the time of addition of ADP is 63% for wild type and 84% for the mutant. To further examine this change in E1P/E2P distribution, an experiment was carried out where the enzyme was phosphorylated at a high Na^+^ concentration of 600 mM, to accumulate as much E1P as possible (cf. Scheme S1), and the dephosphorylation was followed upon addition of 20 mM K^+^ (Fig. 3F). In this case, the rate constant corresponding to the slow phase of dephosphorylation was found 3-fold increased for the mutant relative to wild type. With sufficient K^+^ present to saturate the external sites activating E2P dephosphorylation, the rapid phase reflects the decay of E2P in the reaction E2P→E2+P_i_ (cf. Scheme S1), whereas the slow phase reflects the rate-limiting conversion of E1P to E2P [36]. The increased rate observed for the mutant may either reflect a true increase in the rate constant of the E1P→E2P conversion or a reduction of the rate constant of the reverse E2P→E1P conversion. The larger amplitude observed for the mutant corresponds to the larger amount of E2P accumulated in the mutant. Overall it may be concluded that the distribution of E1P and E2P is shifted toward E2P in the mutant, relative to the wild type, and that the reason may be an enhanced E1P→E2P transition rate or a reduced E2P→E1P rate. Because the E1P→E2P transition involves the release of Na^+^, and E2P can only be transformed back to E1P with Na^+^ bound (Scheme S1), a possible explanation is that Na^+^ binds with reduced affinity not only from the cytoplasmic side, but also from the extracellular side, and that Na^+^ binding in E1P is destabilized in the mutant. Because E1P is a Na^+^-occluded state, which is stabilized by the docking of the C-terminus between the transmembrane segments [31], [32], it seems likely that the interaction of the arginine with the C-terminus helps positioning the C-terminus correctly for stabilizing the occluded state.

### Identification of the porcine *ATP1A2* and the *ATP1A3* coding sequences

Our cloned porcine *ATP1A2* coding sequence consists of an open reading frame of 3060 bp and flanking regions (GenBank ID: GQ340776). Alignment with the previously published *ATP1A2* coding sequence (GenBank ID: NM_001171541/ [37]) reveals complete identity. The deduced amino acid sequence (1020 amino acid residues, sequence not shown) differs from its human counterpart in nine positions (pig/human): 69V/I; 170V/I; 496 N/S; 517 S/T; 540 L/M; 649 V/M; 734 A/S; 836 P/S and 891 S/T.

**Figure 4:**
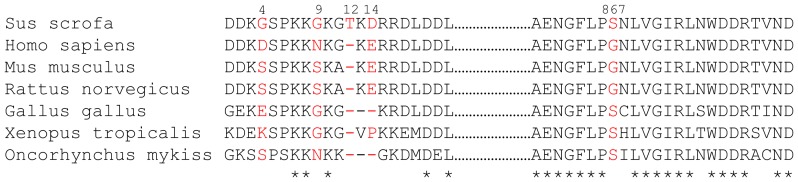
Comparison of the porcine Na^+^/K^+^-ATPase α3 amino acid sequence with α3-sequences from other species. Pig (Sus scrofa, GQ340775), human (Homo sapiens, NM152296), mouse (Mus musculus, BC037206), rat (Rattus norvegicus, NM012506), chicken (Gallus gallus, NM205475), frog (Xenopus laevis, 001086971), and rainbow trout (Oncorhynchus mykiss, NM001124630). The amino acids indicated in red show the five positions where porcine and human sequences differ. The numbering of the residues refers to the porcine sequence (the first five residues removed by posttranslational modification are not numbered). * indicates identity in all seven species.

Our cloned porcine *ATP1A3* coding sequence consists of an open reading frame of 3042 bp and the flanking regions (GenBank ID: GQ340775). Compared with the sequence of human *ATP1A3* cDNA (GenBank ID: NM_152296), encoding a protein of 1013 residues, the pig *ATP1A3* cDNA sequence was found to be three base pairs longer, encoding an extra amino acid, threonine, at position 12 (Fig. 4). The presence of this extra amino acid was confirmed by examination of the genomic porcine *ATP1A3* sequence [38]. Apart from the inserted threonine at position 12, the porcine α3-protein differs at four positions (red in Fig. 4) from its human counterpart of 1013 amino acids (>99% amino acid identity). Three of these differences are in the most N-terminal part, and one is at position 867 in the extracellular loop between transmembrane segments M7 and M8. Only one of the replacements, aspartate for glutamate at position 14, is conservative. The N-terminus is one of the most variable regions between the isoforms, and is generally well conserved across species for the same isoform [9]. However, the N-terminal positions of variation between pig and human α3 do not belong to those generally showing highest conservation across species. Worth noting is also that both of our cloned α2 and α3 porcine sequences like their human counterparts contain the arginine homologous to α1 Arg841 shown above to be important for Na^+^ binding.

### Chromosomal localization of porcine *ATP1A1*, *ATP1A2*, and *ATP1A3*

The PCR results obtained for the three genes using the porcine-rodent somatic cell hybrid panel were analyzed using the IMpRH mapping tool (http://imprh.toulouse.inra.fr/). This suggested a chromosomal localization of *ATP1A1* and *ATP1A2* to porcine chromosome 4, which is in agreement with previous studies reporting *ATP1A1* to be localized to porcine 4q16-q23 and *ATP1A2* to be localized to porcine 4q21-q23 [39], [40]. We found *ATP1A1* linked to SW2435 (LOD score 4.01, distance 0.72 ray) and *ATP1A2* linked to SW589 (LOD score 11.64, distance 0.26 ray). The genes *ATP1A1* and *ATP1A2* have both been mapped to the human chromosome 1, region 1p21-cen and region 1cen-q32, respectively [41], while the *ATP1A3* gene was found linked to markers on chromosome 19q13.2 [42], [43]. We localized *ATP1A3* to the porcine chromosome 6, linked to SW133 with a LOD score of 13.37 and a distance of 0.16 ray. The marker SW133 was localized to the porcine chromosome 6q12, in accordance with the map found using the web site (http://www.marc.usda.gov/genome/swine/htmls/Chromosome06.html/). Recently, new data from the porcine genome sequencing has allowed us to use Blat software to localize the *ATP1A1*, *ATP1A2*, and *ATP1A3* genes in the Sus scrofa 10.2 genome [38]. The *ATP1A1* gene maps to SsChr4:114,432,308–114,451,893, *ATP1A2* maps to SsChr4:98,284,935–98,307,276, and *ATP1A3* is localized on chromosome 6:45,929,172–45,946,082 (Table 1) confirming our observations.

**Table 1:** Methylation status of the *ATP1A1*, *ATP1A2*, and *ATP1A3* genes in pig brain and liver (Sus scrofa 10.2).

Gene	Length (bp)	Chr	Start	End	Tissue	Methylated reads	Total reads	Methylation percentage (%)
					Brain	4,330	5,663	**76.5**
ATP1A1	3,083	4	114,432,308	114,451,893				
					Liver	12,403	16,092	**77.1**
					Brain	3,737	5,527	**67.6**
ATP1A2	3,082	4	98,284,935	98,307,276				
					Liver	14,094	21,463	**65.7**
					Brain	2,725	4,041	**67.4**
ATP1A3	3,066	6	45,929,172	45,946,082				
					Liver	7,340	12,711	**57.7**
					Brain	0	81	**0**
pATP1A3	487	6	45,818,731	45,819,217				
					Liver	21	1,507	**1.4**

A selected promoter region of the *ATP1A3* (pATP1A3) gene was also included in this study.

### Methylation status of the *ATP1A1*, *ATP1A2*, and *ATP1A3* genes

The methylation status of the *ATP1A1*, *ATP1A2*, and *ATP1A3* genes was examined by whole-genome bisulfite sequencing of DNA isolated from pig liver and brain (occipital cortex). Data for this analysis are shown in Table 1. The methylation level of the *ATP1A1* gene, covering approx. 20 kb, was approx. 77% in both pig liver and brain. A lower methylation level, around 67%, for both liver and brain was found for the *ATP1A2* gene, covering 22.3 kb. For the *ATP1A3* gene, approx. 20 kb of the sequence, including 22 of 23 exons and intervening introns, was analyzed for methylation. In occipital cortex, the methylation level was 67.4%, and in liver a methylation level of 57.7% was found. Because we have analyzed tissue samples with heterogeneous populations of cells, the methylation status of a particular CpG could range from 0% to 100%. The methylation status for each CpG in a region or gene expressed as a frequency is often classified into different categories as proposed by Laurent et al (2010) [44]. According to this classification the methylation status for the entire coding region of *ATP1A3*, *ATP1A2*, and *ATP1A1* is intermediate between partially methylated and methylated (M_P: 60–80%). Even though the methylation levels of *ATP1A3* in the liver and brain are in the same category, more detailed investigation of single CpGs showed that there are 54 CpGs that are differentially methylated in the two tissues. Of these, 19 are located in exonic regions, and the remaining are in intronic regions. Differentially methylated CpGs in exon 1 of this gene showed higher methylation in liver (>60% difference between liver and brain). Conversely, in exon 12 the CpGs showed higher methylation in brain. It has been demonstrated that methylation surrounding the downstream region of the transcription start site (TSS) is tightly linked to transcriptional silencing [45]. Accordingly, we found the first exon of *ATP1A3* in liver methylated, which might explain the lack of expression of this gene in liver.

A 487 bp DNA stretch in the *ATP1A3* promoter (see below) was also examined for methylation. Only 21 CpGs of 1507 were found methylated in liver, yielding a methylation degree of 1.4%, i.e. very low. No methylated CpGs were identified in brain (occipital cortex). According to the classification [44] this means an unmethylated status (U:<20%). It is estimated that between 60% and 80% of all CpGs are methylated in mammals [46], [47]. Unmethylated CpGs are often clustered in CpG islands (CGIs) in promoter regions of mainly house-keeping genes [48]. Saxonov et al. 2006 [49] estimated that approx. 70% of promoters belong to a class with high CpG content and around 30% are in a class with a CpG content characteristic of the overall genome i.e. a low CpG content. The linkage between gene promoter methylation and transcriptional suppression has been well recognized for several years. In general, genes with hypermethylated promoters are transcriptionally silent, and DNA methylation gradually accumulates upon long-term gene silencing [45]. In some cancers promoter CGIs become hypermethylated resulting in transcriptional silencing of tumor suppressor genes. From our data we conclude that the lack of expression of *ATP1A3* in liver is not the result of methylation of the liver promoter, whereas it could be the result of methylation of the first exon in liver.

### Relative expression of porcine *ATP1A1*, *ATP1A2*, and *ATP1A3* mRNA

**Figure 5:**
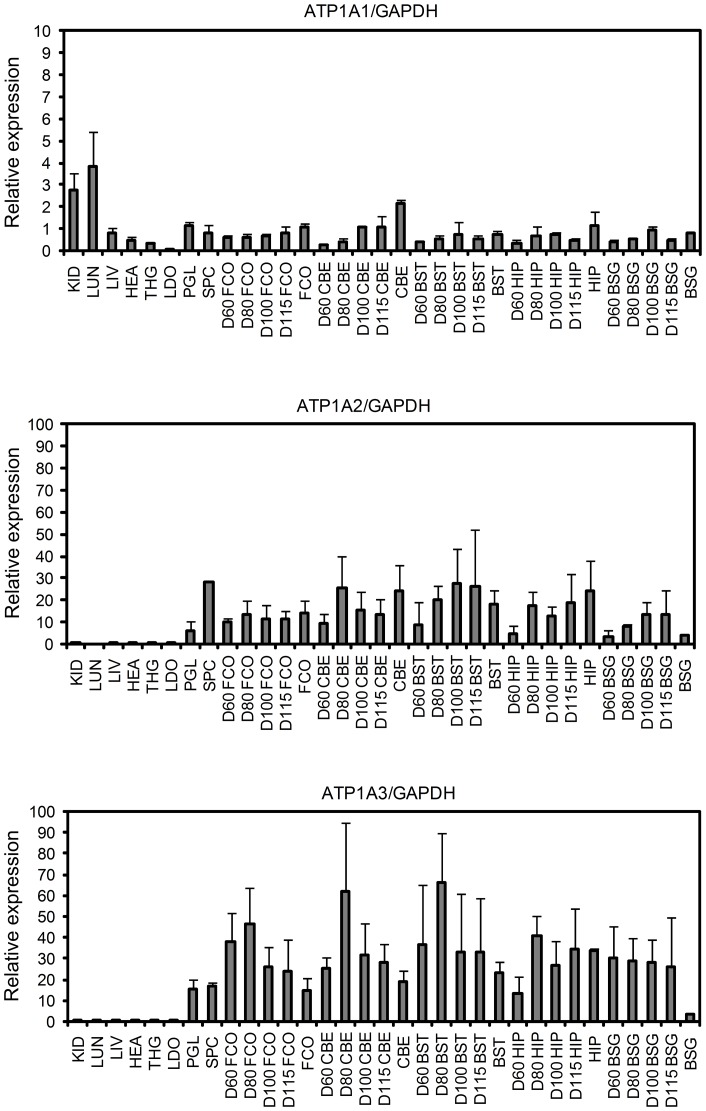
Relative expression pattern of porcine *ATP1A1*, *ATP1A2*, and *ATP1A3* mRNA in different organs and tissues from adult pigs and from brain tissues at different stages of embryonic development. *GAPDH* is used as endogenous reference. Each column represents the mean expression of a triplicate from three different pigs. The considerable biological variation between the animals represented in each column is indicated by error bars showing the standard deviation. KID: kidney, LUN: lung, LIV: liver, HEA: heart, THG: thyroid gland, LDO: longissimus dorsi, PGL: pituitary gland, SPC: spinal cord, FCO: frontal cortex, CBE: cerebellum; BST: brain stem, HIP: hippocampus, BSG: basal ganglia, D60: embryo of day 60, D80: embryo of day 80, D100: embryo of day 100, D115: embryo of day 115.

**Figure 6:**
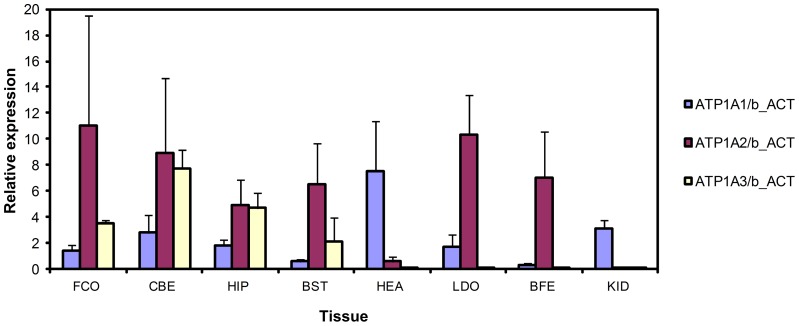
Comparative expression levels of porcine *ATP1A1*, *ATP1A2*, and *ATP1A3* mRNA in different organs and tissues from adult pigs. *β-actin* (b_ACT) is used as endogenous reference. Each column represents the mean expression of a triplicate from three different pigs. The considerable biological variation between the animals represented in each column is indicated by error bars showing the standard deviation. FCO: frontal cortex, CBE: cerebellum, HIP: hippocampus, BST: brain stem, HEA: heart, LDO: longissimus dorsi, BFE: biceps femoris, KID: kidney.

In order to investigate the relative expression pattern of *ATP1A1*, *ATP1A2*, and *ATP1A3* mRNA in different tissues, including brain tissues from four embryonic developmental stages, we performed quantitative real-time analyses. Such a quantitative determination of the relative *ATP1A1*, *ATP1A2*, and *ATP1A3* mRNA levels in a series of tissues has not previously been reported for humans or pig. The analyzed pig organs and tissues include kidney, lung, liver, thyroid gland, heart, longissimus dorsi muscle, biceps femoris muscle, brain, and spinal cord. The brain tissues analyzed include pituitary gland, frontal cortex, cerebellum, brainstem, hippocampus, and basal ganglia. In addition to the brain tissues from adult pigs, brain tissues from porcine embryos sampled at day 60, 80, 100, and 115 of gestation were also analyzed. Day 115 of gestation is the day of farrowing. Three biological samples were included for each tissue and for each time point. The PCR analyses were performed in technical triplicates, and the obtained data were normalized to *GAPDH* expression (Fig. 5) or *β-actin* expression (Fig. 6) in order to compensate for inter-PCR variation due to differences in RNA integrity and sample loading. Note that due to differential expression of the reference genes *GADPH* and *β-actin* the expression levels in Fig. 5 and Fig. 6 are not directly comparable.

*ATP1A1* transcript was detected in all the porcine tissues analyzed. In kidney, lung, liver, and thyroid gland only the *ATP1A1* transcript was found expressed, while the *ATP1A2* and *ATP1A3* transcripts were observed in brain tissues and spinal cord (Fig. 5 and Fig. 6) as also reported for human and other mammals [11], [13], [50], [51]. Furthermore, *ATP1A2* transcript is the predominant isoform found in skeletal muscle, longissimus dorsi, and biceps femoris, and a very low level of *ATP1A2* expression was observed in heart (Fig. 6). We did not observe any expression of *ATP1A3* mRNA in the heart. This is in contrast to the human heart, where all the three isoforms α1, α2, and α3 are found expressed [16]–[18]. In this respect pig heart resembles the adult rat heart, where only α1 and α2 are expressed. It is well recognized, however, that there is considerable species variation in the expression of α2 and α3 in heart [17]. Expression of α3 in heart has been observed in human, monkey, ferret, and dog, as well as in fetal rat [9]. Worth noting, the switch in isoform expression from α3 to α2 between fetal and adult rat heart coincides with other major changes in cardiac electrophysiology and calcium metabolism [52]. We conclude that except for lack of α3 expression in the heart, the expression pattern observed in pig resembles that found in human.

The expression analysis of the different brain tissues from developing porcine embryos (Fig. 5), revealed the presence of *ATP1A1*, *ATP1A2*, and *ATP1A3* transcripts in the frontal cortex, cerebellum, brainstem, hippocampus, and basal ganglia as early as 60 days of gestation as well as at 80, 100, and 115 days of gestation. These four time points of gestation cover important stages in brain development. For example, convolution of the porcine brain is reported to take place during the period from day 60 to day 80 [53]. The presence of all three Na^+^/K^+^-ATPase transcripts in the porcine brain during embryonic development, indicate that the Na^+^/K^+^-ATPase α1-, α2-, and α3-isoforms are important, not only for the adult brain function but also for embryonic brain development. Notably, a major biological variation was observed for all three genes, especially *ATP1A2* and *ATP1A3* (shown by the standard deviations in Fig. 5 and Fig. 6), indicating that the expression levels of *ATP1A2* and *ATP1A3* transcript differ markedly between individuals.

### Western blot analysis

**Figure 7:**
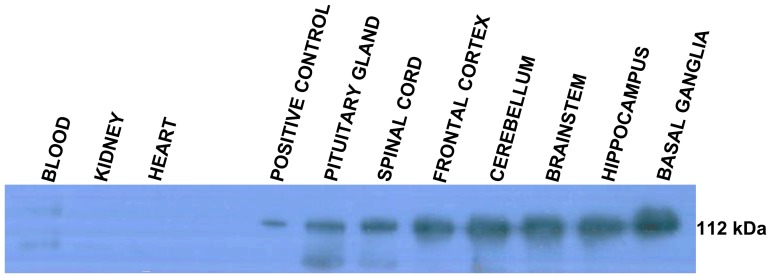
Western blot showing the α3-isoform specifically expressed in porcine neural tissues. An α3-specific antibody reactive band of approximately 112 kDa corresponding to Na^+^/K^+^-ATPase is present in all brain tissues analyzed and in the spinal cord. Cell lysate from COS-1 cells stably expressing *ATP1A3* was used as positive control.

The highly specific expression of the *ATP1A3* transcript in neuronal tissue prompted us to determine the expression of the α3-isoform also at protein level. Western blot analysis was performed with total protein isolated from adult pig brain tissues (pituitary gland, frontal cortex, cerebellum, brainstem, hippocampus, and basal ganglia) and tissues from spinal cord, kidney, and heart. Also, porcine blood was analyzed (Fig. 7). To compensate for biological variation, each protein sample was composed of a pool of equal concentrations of proteins from three different animals. The positive control consists of protein lysates from COS-1 cells transfected with human *ATP1A3*. The blot showed presence of the Na^+^/K^+^-ATPase α3-isoform in porcine brain tissues and spinal cord (Fig. 7), while no protein expression was observed in blood, kidney, and heart, thus confirming our results for *ATP1A3* at transcript level. Our results for Na^+^/K^+^-ATPase α3-polypeptide expression in pig are also in agreement with data published for other species, as expression of the α3-isoform was reported in various brain tissues, while no α3 expression was found in kidney of human, rat, sheep, chicken, and guinea pig [9].

### Cloning of pig *ATP1A3* promoter

**Figure 8:**
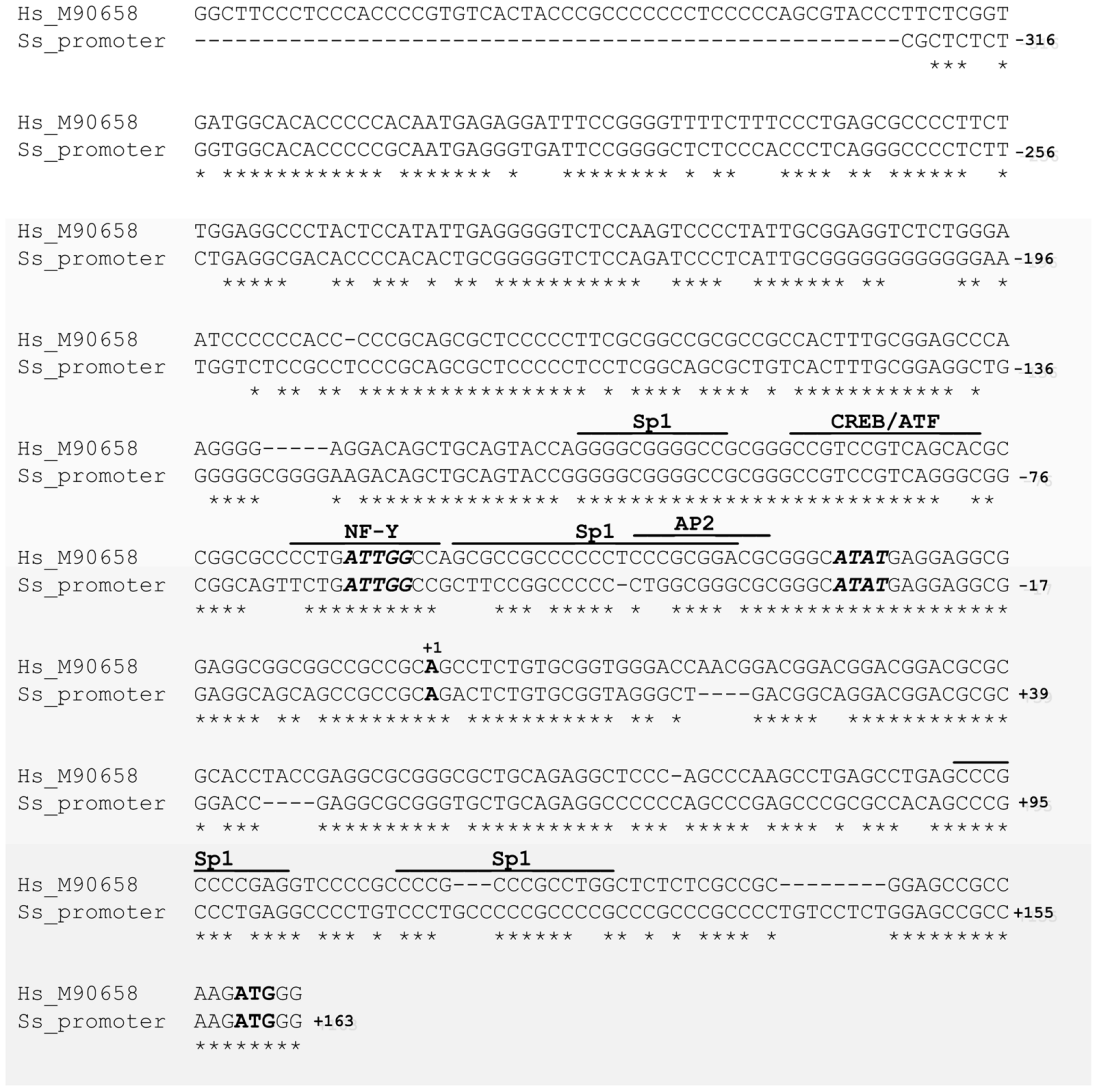
Comparison of the cloned porcine *ATP1A3* promoter with the human *ATP1A3* promoter. Nucleotides are numbered with reference to the putative transcriptional start site (+1). Lines indicate putative transcription factor binding sites. The CCAAT and TATA boxes are indicated in bold italics. Bold ATG indicates the start site of translation. Hs: Homo sapiens, Ss: Sus scrofa. Following the abbreviation of the species, the accession numbers are shown.

As a basis for understanding the factors governing the tissue specific expression of the porcine Na^+^/K^+^-ATPase α3-isoform, we have for the first time structurally and functionally characterized the putative promoter region of the porcine *ATP1A3* gene. The 5′-region immediately flanking the translation start site of *ATP1A3* was cloned from porcine genomic DNA by PCR, and the promoter sequence has been assigned the GenBank accession no. JQ413416. The PCR amplified product consists of 488 bp (Fig. 8), and by applying a computer-assisted analysis (http://www.cbrc.jp/research/db/TFSEARCH.html) of the 488 bp sequence, several putative transcription factor binding sites were revealed. To identify conserved DNA sites, the porcine *ATP1A3* promoter was compared to the sequence of the human *ATP1A3* promoter (GenBank accession no. M90658) [54], [55] as shown in Fig. 8. Benfante and colleagues [55], describing a human minimal promoter, reported putative binding sites for Sp1 (Specificity protein 1), CREB (cAMP-response-element-binding protein)/ATF (activating transcription factor), the NF-Y (nuclear factor-Y)/C-EBP (CCAAT-enhancer-binding protein), the AP-2 (activator protein 2), and for the TATA-binding protein. Importantly, almost all the binding sites reported for the human promoter are conserved in the porcine *ATP1A3* promoter. The TATA box, the CCAAT box, the putative CREB/ATF binding site and the Sp1 binding site just upstream of the CREB/ATF-binding site are completely conserved between pig and man (Fig. 8), suggesting these binding sites to be important for activity of the promoter. By contrast, only the TATA box and the CCAAT box are completely conserved between human and rat *ATP1A3* promoters [54].

### Analysis of the porcine *ATP1A3* promoter in zebrafish

**Figure 9:**
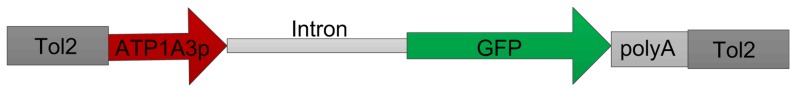
Schematic representation of elements in the minimal *Tol2*-vector construct, Tol2-pATP1A3:GFP, used for transgenesis in zebrafish. This construct is modified from the pT2AL200R150G plasmid [70]. Tol2: the left and right terminal regions of the full-length Tol2, ATP1A3p: the porcine *ATP1A3* promoter sequence, GFP: Green Fluorescence Protein, Intron: the rabbit β-globin intron, polyA: SV40 polyA signal.

To assess the activity and specificity of the cloned porcine *ATP1A3* promoter in a whole organism harboring the complexity of developmental and tissue-specific gene regulation, we took advantage of the zebrafish model. We used the *Tol2* transposon system [56] to produce transgenic zebrafish embryos expressing GFP driven by the porcine *ATP1A3* promoter (the *Tol2*-vector construct is shown in Fig. 9).

**Figure 10:**
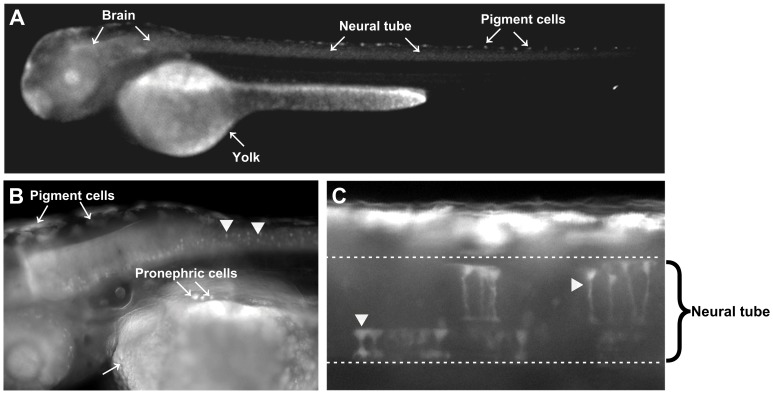
Specificity of *ATP1A3* promoter driven expression of GFP in zebrafish embryos. **A.** and **B.** Weak GFP expression in the central nervous system and cells of the pronephros in F1 embryo 54 hours post fertilization. **C.** Mosaic expression in individual cells of the neural tube driven by the *ATP1A3* promoter in a representative embryo of the injected generation.

Embryos co-injected with Tol2 transposase-encoding mRNA and Tol2-ATP1A3p:GFP plasmid were reared to sexual maturity and crossed to wild type for assessment of specific promoter activity in the F1 generation. Four of six adults were fertile, and three of those produced GFP-positive offspring as determined by fluorescence microscopy (Table 2). All GFP positive F1 embryos showed weak expression in the central nervous system (CNS) and in cells of the pronephros (Fig. 10A and B). However, a fraction of GFP positive embryos showed additional expression in the pharyngeal arches (Founder 2) or notochord (Founder 3), suggesting transgene insertion near regulatory sequences directing expression in these tissues. CNS expression was detected at all assessed stages throughout embryonic and larval development from as early as 24 hours post fertilization. Fig. 10C shows mosaic expression in individual neural tube cells of an injected embryo driven by the *ATP1A3* promoter. These data strongly suggest that the cloned porcine *ATP1A3* promoter is active in the CNS and cells of the pronephros, and that the promoter is specific for these tissues in zebrafish.

**Table 2:** GFP expression patterns in F1 embryos evaluated by fluorescence microscopy.

	Total	GFP neg.	GFP pos.	CNS	Pronephros	Notochord	Pharyngeal arches
Founder 1	144	98	46	100%	100%	0%	0%
Founder 2	142	112	30	100%	100%	0%	46.7%
Founder 3	63	37	26	100%	100%	38.5%	0%

Absolute numbers of GFP–positive and –negative embryos are given, and tissues of expression with percent of total GFP positive embryos are specified.

The observed *ATP1A3* promoter activity in the central nervous system of zebrafish is in line with the preferential expression of the Na^+^/K^+^-ATPase α3-isoform in porcine brain and spinal cord (cf. Figs. 5–7) as well as in neuronal tissue of other mammals. The activity of the *ATP1A3* promoter in cells of the pronephros was unexpected, because the expression analyses of porcine kidney showed no expression of the Na^+^/K^+^-ATPase α3-isoform at transcript level or protein level (Figs. 5–7), as has also been reported for human, rat, and bovine kidney [9], [16], [57], [58]. One possible reason for the observed activity of the porcine *ATP1A3* promoter in zebrafish pronephros is that regulatory proteins, such as transcription factors, vary between mammals and fish. However, in the light of our result for the zebrafish pronephros it is of note that some studies using antibodies against α3 to detect expression at protein level have identified α3 reactivity in collecting duct of rabbit kidney [59] and in tubule cell mitochondria from rat kidney [60]. In zebrafish, there are two endogenous orthologs of *ATP1A3*: *atpa3a* and *atpa3b*, which are expressed in neuronal tissue like the mammalian α3. An additional and unique expression of the α3a-ortholog was found in gut, whereas endogenous α3-ortholog expression in the zebrafish pronephros has not been detected [61], [62].

### Conclusions and Perspectives

The overall conclusion of the present study is that there is a high resemblance between pig and human with respect to the amino acid sequences and expression patterns of α1-, α2-, and α3-isoforms of Na^+^/K^+^-ATPase. All three isoforms show >99% amino acid identity between pig and human. Of notice is that the porcine α3-isoform has an insertion of an extra residue (Thr12) relative to the human counterpart. Furthermore, the α1-sequence determined here was found to differ from the previously published α1-sequence at five positions, thereby being in accordance with a high conservation between species at these positions. Structural analysis suggests that one of these five residues, namely Arg841, by electrostatic interaction helps positioning the C-terminus between transmembrane segments in the protein. Our functional analysis of a mutant, R841A, showed that the arginine is important for Na^+^ binding, but not for K^+^ binding, in accordance with a role of the C-terminus in stabilizing the third Na^+^ site. The chromosomal localization of the three porcine *ATP1A1*, *ATP1A2*, and *ATP1A3* genes was determined and the methylation status reported. The first exon of *ATP1A3* is methylated at a higher level in liver than in brain, which might explain the lack of expression of this gene in liver. All three genes were found expressed in various parts of CNS, at 60–115 days of embryonic gestation and in adult pigs. The α3-isoform is confined to neuronal tissue. Of notice is the lack of expression of α3 in the porcine heart, a difference from the human expression pattern identified both at mRNA and protein level. The sequence of the putative promoter region of the porcine *ATP1A3* gene showed complete conservation of the protein binding sites reported for the human *ATP1A3* promoter, which is in contrast to the rat counterpart. In transgenic zebrafish, expression directed from the porcine *ATP1A3* promoter occurred primarily in the CNS, however expression was seen also in the pronephros.

Examples of successful use of transgenic pigs as animal models in studies of human diseases [26], [27] have prompted us to explore the potential of pig as model in studies of the neurological diseases RDP and AHC, caused by mutations of the Na^+^/K^+^-ATPase α3-isoform [20], [24]. Porcine models of RDP and AHC may help to provide information about the underlying cellular and molecular mechanisms of these disorders, and develop more effective treatments. The models would moreover be valuable tools for testing environmental factors that could be implicated in the onset of disease. The pig is a highly valuable experimental animal because of its suitability for surgery, and furthermore its lifespan is much more similar to that of humans than the lifespans of rodents and other experimental animals, thus making pig particularly attractive for studies of disorders like RDP and AHC that develop in an age dependent manner and are precipitated by certain events during the life span. The results in the present study provide a firm basis for studies employing the *ATP1A3* promoter in combination with the cloned Na^+^,K^+^-ATPase cDNA in attempts to generate transgenic pigs overexpressing mutated porcine Na^+^,K^+^-ATPase.

## Methods

### Ethic Statements

The pigs were housed and used in compliance with European Community animal care guidelines. Beforehand, the experimental procedures were approved by the National Ethical Committee in Denmark (Approval No. 2010/561-1891). Pigs were sacrificed by an injection with 30 mg/kg Pentobarbital (Vipidan, Denmark). Experiments involving zebrafish were carried out in accordance with the recommendations from the European network on fish biomedical models and according to Danish legislation. All zebrafish used in this study were under the age of 72 hours, and hence the experiments do not require any approval. Zebrafish embryos were killed by a tricaine overdose.

### Extraction of nucleic acids

The pig tissues used for RT-PCR cloning of *ATP1A1*, *ATP1A2*, and *ATP1A3* and for real-time expression analysis were obtained from adult Danish Landrace pigs. After removal, tissues were dissected at −20°C and total RNA was isolated by use of the mirVana miRNA Isolation kit (Ambion) following the manufacturer's instructions. The integrity of the RNA samples was verified by ethidium bromide staining of the ribosomal RNA on 1% agarose gels.

### cDNA synthesis

Synthesis of cDNA used for cloning was performed with 2 µg of total RNA isolated from pig frontal cortex and cerebellum using SuperScript III Reverse Transcriptase (Invitrogen). The cDNA synthesis was initiated by heating of total RNA, oligo(dT)-primer and dNTP mix at 65°C for 5 min, and the mixture was placed on ice for 1 min, followed by the addition of 200 U SuperScript III reverse transcriptase, 1X first-strand buffer, 5 mM DTT and H_2_O to a final volume of 20 µL. The total mixture was incubated at 42°C for 50 min, followed by 70°C for 15 min.

Synthesis of cDNA used for quantitative real-time RT-PCR was performed with 1 µg of total RNA isolated from various adult porcine organs and tissues, and from embryonic brain tissues sampled at day 60, 80, 100, and 115 of gestation. The latter is the day of farrowing. Random hexamers (Roche) were used as primers in the cDNA synthesis. Total RNA, random hexamers, dNTP mix and H_2_O in a total volume of 13 µL was incubated at 65°C for 5 min, placed on ice for 1 min, followed by the addition of 200 U SuperScript III reverse transcriptase, 1X first-strand buffer, 5 mM DTT and H_2_O to a final volume of 20 µL. The total mixture was incubated at 25°C for 5 min, 42°C for 50 min and 70°C for 15 min.

### Cloning of the porcine *ATP1A1*, *ATP1A2*, and *ATP1A3* coding sequences

In order to isolate the sequences, *ATP1A1*, *ATP1A2*, and *ATP1A3*, the local porcine EST data bank at the Department of Molecular Biology and Genetics, Aarhus University, and the Sus scrofa PreEnsemble database (http://pre.ensembl.org/index.html) were used [38]. Sequence similarity searches were carried out with alignments using the porcine sequence (GenBank ID: BC_003077) for *ATP1A1* and the human sequences (GenBank ID: NM_000702 and GenBank ID: NM_152296) for *ATP1A2* and *ATP1A3*, respectively. The porcine cDNA fragments thus identified were used to derive oligonucleotide primers for PCR cloning. The primer pairs used to amplify the porcine *ATP1A1* and *ATP1A2* cDNAs were ATP1A1-F, ATP1A1-R and ATP1A2-F, ATP1A2-R, respectively (Table S1). The PCR was conducted in a total volume of 10 µL containing 1 µL 10X diluted cDNA synthesized from RNA isolated from frontal cortex or cerebellum, 0.5 µM of each primer, 200 µM dNTP, 1X Phusion HF reaction buffer, 0.02 U Phusion DNA polymerase (Finnzymes) and sterile H_2_O. The PCR program settings for *ATP1A1* were as follows: Initial denaturation for 1 min at 98°C, and 35 cycles at 98°C for 5 sec, 61°C for 20 sec, 72°C for 1 min, followed by a final extension of 72°C for 5 min. For *ATP1A2*, the program settings were as follows: Initial denaturation at 98°C for 1 min followed by 10 cycles at 98°C for 5 sec, touch-down at 68–62°C for 20 sec and 72°C for 1 min; 25 cycles at 98°C for 5 sec, 62°C for 20 sec, 72°C for 1 min, and a final extension of 72°C for 5 min. The degenerate forward primer ATP1A3-F and the reverse primer ATP1A3- R1 (Table S1) were designed and used to amplify a fragment of the 5′- termination of the *ATP1A3* coding sequence. This amplicon was then used to design the three primers, A3SP1-R, A3SP2-R and A3SP3-R (Table S1), used for 5′ RACE (Rapid Amplification of cDNA Ends) (see the section below). The primers ATP1A3-A3F and ATP1A3-R were subsequently designed to amplify the entire coding sequence of porcine *ATP1A3*. The PCR reaction was carried out in a total of 10 µL similar to that described for *ATP1A1* and *ATP1A2*. The PCR program settings were as follows: 98°C for 1 min, 30 cycles at 98°C for 5 sec, 62°C for 20 sec, 72°C for 1 min, and a final extension of 72°C for 5 min.

After each PCR reaction was completed, 3′-A-overhangs were generated by the polymerase DyNAzyme EXT (Finnzymes) of 72°C for 15 min followed by agarose gel electrophoresis of the PCR products. The amplicons of interest were isolated using QIAquick gel extraction kit (Qiagen) and cloned into the pCR2.1-TOPO® vector (Invitrogen). The inserts were completely sequenced in both directions by the dideoxy chain method using the BigDye Terminator version.3.1 cycle sequencing kit (Applied Biosystems) and the automatic 3730xl DNA analyzers (Applied Biosystems). The software program Sequencer version 4.7 (Gene Codes Corporation) was used to analyze all the sequences.

The porcine coding sequences determined were submitted to the GenBank and have been assigned the following accession numbers: *ATP1A1* (GenBank ID: GQ340774), *ATP1A2* (GenBank ID: GQ340776) and *ATP1A3* (GenBank ID: GQ340775).

### 5′ RACE

Because we were unable to identify cDNA fragments corresponding to the translation start site of the porcine *ATP1A3* gene, the missing 5′-sequence was obtained using 5′RACE procedures, after which the primer pairs amplifying the entire coding sequence could be designed. For this purpose the 5′/3′ RACE kit, 2nd generation (Roche) was used. One µg of total RNA isolated from pig frontal cortex and cerebellum, and the primer A3SP1-R (Table 1) was used to carry out the first-strand cDNA synthesis following the manufacturer's instructions. Immediately after the cDNA synthesis a hydrolysis reaction degrading the original RNA was performed as follows: 10 µL 0.1 M NaOH was added to the cDNA followed by 30 min incubation at 70°C, and to neutralize the pH, 10 µL 0.1 M HCl was added. Subsequently, the cDNA was purified by using the Low Elution cDNA purification module from the SuperScript plus direct cDNA labeling system (Invitrogen) following the manufacturer's protocol except that only 500 µL binding buffer was added to the cDNA. The addition of the poly(A^+^) tail to the 3′end of first-strand cDNA was carried out following the manufacturer's instructions (5′/3′ RACE kit, Roche). The mixture for the PCR amplification of dA-tailed cDNA was set up in a total of 20 µL containing 2 µL dA-tailed cDNA, 1.88 µM OligodT-anchor primer (Roche), 0.625 µM of the primer A3SP2-R, 0.4 µM dNTP mix (Roche), 1X Phusion HF reaction buffer, 0.02 U Phusion DNA polymerase (Finnzymes) and sterile H_2_O. The PCR program settings were as follows: Initial denaturation for 30 sec at 98°C and 35 cycles at 98°C for 15 sec, 59°C for 30 sec, 72°C for 1 min and 30 sec, and a final extension at 72°C for 10 min. The second PCR round (nested PCR) was similar to the first PCR round except for the template, which consisted of 1 µL PCR product and the two primers, the PCR anchor primer (Roche) and A3SP3-R primer (Table S1). The PCR conditions were as follows: Initial denaturation 30 sec at 98°C, and 35 cycles at 98°C for 15 sec, 58°C for 30 sec, 72°C for 2 min, and a final extension at 72°C for 10 min. The amplicons of interest were purified from agarose gels by use of QIAquick gel extraction kit (Qiagen). Since Phusion is a proof-reading enzyme creating blunt ends, 3′-A-overhangs were subsequently generated by DyNAzyme EXT (Finnzymes) at 72°C for 15 min, followed by ligation into the pCR2.1-TOPO® vector and sequencing in both directions.

### Mapping of the porcine *ATP1A1*, *ATP1A2*, and *ATP1A3* genes

Chromosomal localization of the *ATP1A1*, *ATP1A2*, and *ATP1A3* genes was determined using the INRA University of Minnesota porcine radiation hybrid (IMpRH) panel [63], [64]. The primer pairs, HBA1-F and HBA1-R (Table S1), used for *ATP1A1* were designed to amplify a 192 bp genomic fragment of exon 4. The primers used in mapping of the porcine *ATP1A2* and *ATP1A3* were; HBA2-F and HBA2-R, HBA3-F and HBA3-R (Table S1), which amplified fragments of exon 4 of both genes consisting of 194 bp and 200 bp, respectively.

The PCR reaction for *ATP1A1* was performed in a total volume of 10 µL containing 2.5 ng panel DNA, 1.0 µM of each primer, 250 µM dNTP, 1X Phusion HF reaction buffer, 0.02 U Phusion DNA polymerase (Finnzymes) and sterile H_2_O. The PCR reaction was conducted using the following conditions: 98°C for 1 min; 10 cycles at 98°C for 5 sec, touch-down annealing 65–60°C for 20 sec and 72°C for 1 min; 25 cycles at 98°C for 5 sec, 60°C for 20 sec and 72°C for 1 min and finally 72°C for 5 min.

The polymerase used for *ATP1A2* and *ATP1A3* was Ampli Taq Gold (Applied Biosystems). For both genes, the PCR was set up in a total volume of 10 µL containing 2.5 ng panel DNA, 1.0 µM of each primer, 250 µM dNTP, 1X PCR reaction buffer II (Applied Biosystems), 2,5 mM MgCl_2_, 0.5 U Ampli Taq Gold polymerase (Applied Biosystems) and sterile H_2_O. The PCR reactions were conducted as follows: Initial denaturation at 94°C for 12 min; 10 cycles at 94°C for 15 sec, touch-down 65–60°C for 15 sec and 72°C for 15 sec; 35 cycles at 94°C for 15 sec, 60°C for 15 sec, and a final extension at 72°C for 15 min, and finally 72°C for 7 min. The PCR results from all three genes were analyzed by use of the web-site tool (http://imprh.toulouse.inra.fr/).

### Real-time expression analysis

Quantitative real-time RT-PCR analysis of porcine *ATP1A1*, *ATP1A2*, and *ATP1A3* mRNA was performed on cDNA synthesized as described in section “cDNA synthesis”. The detection system used was the ABI Prism 7900HT sequence detection system (Applied Biosystems). A 5′nuclease assay was employed, using the probes #79(*ATP1A1*), #42(*ATP1A2*), and #85(*ATP1A3*) from the Universal Human ProbeLibrary (Roche) and the three primer pairs A1RT-F and A1RT-R2 (*ATP1A1*), A2RT-F2 and A2RT-R (*ATP1A2*), A3RT-F and A3RT-R2 (*ATP1A3*) (Table S1). All probes from the Universal Human ProbeLibrary were labelled with the fluorescent reporter SYBR Green. The *ATP1A1*-, *ATP1A2*-, and *ATP1A3*-specific primers were designed to span the exon 16 - exon 17 junction, the exon 7 - exon 8 junction and the exon 19 - exon 20, respectively. The mixture for the PCR reactions contained: 2 µL cDNA (undiluted, 2X, 10X, 25X, or 100X diluted depending on the gene and the tissue analyzed), 0.3 µM forward primer, 0.3 µM reverse primer, 0.125 µM probe, 5 µL Taq-Man Master Mix (Applied Biosystems), and H_2_O to obtain a final volume of 10 µL. The reactions were run at the default setting program i.e., initial denaturation and polymerase activation of 50°C for 2 min and 95°C for 10 min followed by 40 cycles at 95°C for 15 sec and annealing/extension at 60°C for 1 min. Glyceraldehyde-3-phosphate dehydrogenase (*GAPDH*) or *β-actin*, depending on the tissues analyzed, were used as endogenous reference. For *GAPDH* and *β-actin*, the mixture for a 10 µL reaction contained: 2 µL cDNA (*GAPDH*: 25X diluted, *β-actin*: 20X diluted), 0.3 µM of each primer (*GAPDH*: GAPDH-F and GAPDH-R, *β-actin*: b_ACT-F and b_ACT-R, Table S1) 0.125 µM probe (GAPDH-probe or b_ACT-probe #56), Table S1), 5 µL Taq-Man Master Mix and H_2_O. The reaction was run at the default settings. All samples, standards and no template controls were amplified in triplicate. Furthermore, to increase the likelihood that the results are biologically significant, tissues from three different animals were analyzed for each type of tissue and organ. For quantification of gene expression changes in organs and tissues, the ΔΔCt method was used to calculate relative fold changes normalized against *GAPDH* or *β-actin*, as described in the user bulletin #2 (Applied Biosystems). The data obtained from real-time quantitative PCR were initially processed in SDS 2.2 and further by use of Microsoft software program Excel.

### Western blot analysis

Total proteins from various porcine organs/tissues were purified using the following procedure: for each sample, a total of 200 mg tissue from three different animals was weighed and mixed with 1,000 µL 1x extraction buffer (50 mM Tris-base pH 8.3, 10 mM EDTA) containing protease inhibitors (Complete, mini, EDTA-free, Roche) followed by homogenization using a tissuelyser (Qiagen). Immediately after homogenization the samples were placed on ice. Another 1,000 µL 1x extraction buffer was added to each sample. The mixture (500 µL) was added to a tube containing 500 µL 2x treatment buffer (0.5M Tris pH 6.8, 10% SDS, 100% Glycerol). The samples were incubated at 50°C for 20 min, mixed and further incubated at 50°C for 5 min. Finally, the samples were centrifuged at 13,000 rpm in 20 min (at room temperature), and the supernatants containing the total protein were transferred to new tubes. Protein concentrations were determined using the bicinchoninic acid method (Pierce BCA assay kit, Bie & Berntsen, Denmark).

Prior to SDS-PAGE, the various protein samples and the positive control (protein lysates from COS-1 cells stably expressing human *ATP1A3*) were incubated at 37°C for 20 min in loading buffer (50 mM Tris-HCl pH 6.8, 4% SDS, 40% glycerol, 20 mM dithioerythreitol, bromophenolblue). PAGE was carried out with the various tissue samples (15 µg total protein) in a 10% Tris-HCl polyacrylamide gel (Ready Gels, Bio-Rad) in parallel with 15 µL protein marker (Prestained Protein size marker, Cell Signaling Technologies), pre-heated to 95°C for 5 min, and 25 µg positive control. Following electrophoresis, proteins were electrophoretically transferred by semi-dry blotting (112V, 2 hours) to a PVDF membrane (Bio-Rad) pretreated by soaking in methanol for 1 min. Subsequently, the membrane was blocked in 5% non-fat dry milk in TBST (20 mM Tris-HCl pH 7.6, 150 mM NaCl, 0.05% Tween 20) overnight at 4°C. Next day, the membrane was incubated for 1 h with mouse monoclonal anti-Na^+^/K^+^-ATPase α3-subunit (A-273, Sigma) diluted (1∶1,000) in TBST, followed by thorough washing in TBST. Immune complexes were detected by chemiluminescence (BM Chemiluminescence Blotting substrate, POD, Roche) after incubation for 30 min with HRP-conjugated polyclonal goat anti-mouse secondary antibody (Dako) diluted (1∶2,000) in TBST and washing with TBST.

### Cloning of the porcine *ATP1A3* promoter

To isolate the sequence of the porcine *ATP1A3* promoter, the local porcine EST data bank at the Department of Molecular Biology and Genetics, Aarhus University, and the Sus scrofa PreEnsemble database (http://pre.ensembl.org/index.html) were screened. The porcine *ATP1A3* promoter sequence was PCR amplified using genomic DNA isolated from Landrace pigs and the primer pairs PA3-F11 and PA3-EX1R1 (Table S1). The reaction mixture contained 80 ng genomic DNA, 0.3 µM of each primer, 200 µM dNTP, 1X Herculase II reaction buffer (Stratagene), 0.2 µL Herculase II fusion DNA polymerase (Stratagene) and H_2_O in a total volume of 10 µL. The PCR program settings were as follows: Initial denaturation for 2 min at 95°C, followed by 10 cycles at 95°C for 20 sec, touch-down annealing at 63–58°C for 20 sec, 72°C for 30 sec, 20 cycles at 92°C for 20 sec, 58°C for 20 sec, 72°C for 30 sec, and a final elongation step at 72°C for 3 min. The 3′-A-overhangs were generated by DyNAzyme EXT (Finnzymes) at 72°C for 15 min. After gel electrophoresis, the amplicon of interest was purified, cloned and sequenced.

### Methylation status of Na^+^/K^+^-ATPase α-isoforms

In brief, the methylation status of Na^+^/K^+^-ATPase α-isoforms were performed by library preparation, sequencing, mapping and analysis. DNA from each sample was extracted and sheared to a size of 200–300 bp using the Covaris Adaptive Focused Acoustics™ (AFA) process (Covaris). Double-stranded DNA fragments were end repaired, A-tailed, and ligated to methylated Illumina adaptors. Ligated fragments were bisulfite converted using the EZ-DNA Methylation-Kit (Zymo research). Following PCR enrichment, fragments of 325 to 425 bp were size selected and sequenced using Hiseq 2000 Illumina sequencing system. We used Novoalign short read aligner (version 2.07.12 http://www.novocraft.com/) to align reads to a reference genome. Novomethyl (Beta.8.0 http://novocraft.com/main/page.php?s=novomethyl) was used to call the consensus sequence, identify cytosines and call their methylation state or percentage of cytosines methylated. For finding the methylation percentage of special genes or sequences from our methylome data file, we used Tabix [65]

### Analysis of mutant rat enzyme stably expressed in COS cells

Using the QuickChange Site-Directed Mutagenesis kit (Stratagene), the mutation corresponding to pig R841A was introduced into the full-length cDNA encoding the ouabain resistant rat α1-isoform of Na^+^/K^+^-ATPase (R843A). The mutant and wild-type rat enzymes were expressed in COS-1 cells obtained from the American Type Culture Collection (ATCC) using the ouabain selection methodology [33], [66]. This methodology relies on the more than 100-fold difference in ouabain sensitivity between the endogenous COS-1 cell Na^+^/K^+^-ATPase and the exogenous Na^+^/K^+^-ATPase, allowing stable cell lines expressing the exogenous enzyme to be isolated in the presence of ouabain, due to preferential inhibition of the endogenous Na^+^/K^+^-ATPase. The stable integration of the mutant cDNA into the genome of the cells was verified by sequencing of the genomic DNA.

For functional analysis, a crude plasma membrane fraction was prepared by differential centrifugation of a homogenate of transfected COS-1 cells grown in medium containing ouabain [66]. The plasma membranes were made leaky by treatment with sodium deoxycholate, and the Na^+^/K^+^-ATPase activity was determined by following the liberation of P_i_ by the Baginski method over a period of 10 min at 37°C [66]. Leaky membranes (10 µg of total protein) were assayed in 500 µL A-medium (30 mM histidine (pH 7.4), 3 mM MgCl_2_, 1 mM EGTA, 10 µM ouabain), to which had also been added various concentrations of NaCl, KCl, ATP, and the inhibitor vanadate to allow determination of the apparent affinities for the ligands as previously described [32], [34]. The presence of 10 µM ouabain ensured that the endogenous COS-1 cell Na^+^/K^+^-ATPase was completely inhibited. The activity was also determined in the presence of 10 mM ouabain, which inhibits the endogenous as well as the expressed exogenous Na^+^/K^+^-ATPase. To calculate the activity referable to the expressed exogenous enzyme, the background ATPase activity measured with 10 mM ouabain was subtracted from the activity measured in presence of 10 µM ouabain.

For studies of the Na^+^ dependence of phosphoenzyme formation, the leaky membrane suspension (10 µg total membrane protein pre-incubated with ouabain to inhibit the endogenous enzyme) was incubated for 10 s at 0°C with 2 µM [γ-^32^P]ATP in 100 µL P-medium (20 mM Tris (pH 7.4), 3 mM MgCl_2_, 1 mM EGTA, 10 µM ouabain), to which 20 µg of oligomycin/mL had been added (to stabilize the phosphoenzyme) together with various concentrations of NaCl and *N*-methyl-d-glucamine (to maintain the ionic strength upon variation of NaCl content). The background phosphorylation was determined in the presence of 50 mM KCl without NaCl [31], [32], [34]. To follow the dephosphorylation, the leaky membrane suspension was phosphorylated in a similar way in 100 µL P-medium without oligomycin, and dephosphorylation was initiated by addition of 1 mM non-radioactive ATP in a chase medium. For E1P/E2P distribution experiments, the NaCl concentration was 20 mM, and the chase medium contained in addition to non-radioactive ATP also 2.5 mM ADP. For E1P→E2P interconversion experiments, the NaCl concentration was 600 mM, and the chase medium contained in addition to non-radioactive ATP also 20 mM KCl [32], [36].

Acid quenching for determination of the amount of phosphorylated Na^+^/K^+^-ATPase protein was performed either directly after the phosphorylation period or at serial time intervals following the addition of the chase solution to follow the dephosphorylation. During phosphorylation and dephosphorylation, the reaction mixture was stirred continually by a vertically orientated tiny magnet bar (8 mm×1 mm) contained in an Eppendorf tube immersed in icewater (0°C), and the ice-cold chase and quench solutions were added manually, timing being controlled by an electronic metronome.

In all phosphorylation experiments, the acid-precipitated ^32^P-labeled phosphoenzyme was washed by centrifugation and subjected to SDS-polyacrylamide gel electrophoresis at pH 6.0 [32], [36], and the radioactivity associated with the separated Na^+^/K^+^-ATPase band was quantified by “imaging” using a Packard Cyclone™ Storage Phosphor System.

The data points shown in the graphs are average values corresponding to 3–8 independent determinations. Nonlinear regression analysis of the data was carried out as previously described [36], [67] using the Sigmaplot program (SPSS, Inc.), and the best fits are shown as lines in the figures. To determine the apparent ligand affinities for Na^+^, K^+^, and ATP (*K*_0.5_ values, i.e. ligand concentrations giving half maximum activation), the Na^+^ dependency of the phosphorylation level and the K^+^ and ATP dependencies of the ATPase activity were analyzed by applying a modified Hill equation, *V* = (*V*_max_−*V*_0_)[*L*]*^n^*/(*K*_0.5_ *^n^*+[*L*]*^n^*)+*V*_0_, where *V* represents the ATPase activity or phosphorylation level, *V*_max_ its maximum value attained at infinite ligand concentration, and *V*_0_ the value in the absence of the ligand. [*L*] is the ligand concentration and *n* the Hill coefficient.

The vanadate dependency of the ATPase activity was analyzed by applying a Hill equation for inhibition, *V* = ((*V*_max_−*V*_∞_)−(*V*_max_−*V*_∞_)[*L*]*^n^*/(*K*_0.5_ *^n^*+[*L*]*^n^*))+*V*_∞_, where *V*_max_ is the activity in the absence of inhibitor, *V*_∞_ the activity of the maximally inhibited enzyme, and *K*_0.5_ is the vanadate concentration giving half maximum inhibition (apparent vanadate affinity).

The dephosphorylation time courses were fitted by the sum of two exponentials, % phosphorylation = (100%−*α*)⋅e^−*kt*^+*α*⋅e^−*ht*^, where *k* and *h* represent the rate coefficients of the rapid and slow components, respectively, of the decay curves and *α* the extent of the slow component.

### Handling of zebrafish

Zebrafish of the AB strain were obtained from the Tübingen zebrafish stockcenter. The fish were fed twice a day and kept at 28.5°C on a 14 hours light/10 hours dark cycle. Embryos were obtained by natural crosses, reared in E3 buffer (5 mM NaCl, 0.17 mM KCl, 0.33 mM MgSO_4_, 10^−5^% methylene blue, 2 mM Hepes pH 7.0), and staged according to Kimmel et al (1995) [68]. Upon completion of gastrulation, E3 buffer was supplemented with 0.003% *N*-phenylthiourea (PTU) (SIGMA) to inhibit pigmentation.

### Construction of Tol2-pATP1A3:GFP plasmid

The Tol2-ATP1A3promoter:GFP (Tol2-pATP1A3:GFP) plasmid was constructed based on the pT2AL200R150G vector kindly provided by Koichi Kawakami, National Institute of Genetics, Japan [69], [70]. The restriction enzymes *Xho*I and *Hin*dIII (New England BioLabs) were used to replace the EF1a promoter, originally present in the pT2AL200R150G vector, with the porcine *ATP1A3* promoter. Primers (XhoI_A3pro-F and HindIII_A3pro-R, shown in Table S1) were designed to add the *Xho*I and *Hin*dIII sites to the ends of the porcine *ATP1A3* promoter sequence by use of PCR. The PCR was performed in a total volume of 10 µL containing 100 ng DNA (pCR2.1-TOPO® Vector (Invitrogen) containing the porcine *ATP1A3* promoter sequence), 1X PCR buffer, 0.02 U Phusion polymerase, 0.25 mM dNTP and 0.5 µM of each primer under the following conditions: Initial denaturing at 98°C for 1 min, followed by 10 cycles at 98°C for 5 sec, touch-down from 67–62°C for 10 sec and extension at 72°C for 10 sec, followed by 25 cycles at 98°C for 5 sec, 62°C for 10 sec, 72°C for 10 sec, and the final extension step for 5 min at 72°C. 3′-A-overhangs were generated by DyNAzyme EXT (Finnzymes) at 72°C for 15 min. Amplified PCR fragments were purified from agarose gels and cloned into pCR2.1-TOPO vector (Invitrogen) followed by DNA purification and sequencing. The pT2AL200R150G-vector and the pCR2.1-TOPO-vector containing *ATP1A3* promoter were digested with *Xho*I and *Hin*dIII. Fragments of interest were purified from agarose gels and the porcine *ATP1A3* promoter was subcloned into *Tol2*-vector, pT2AL200R150G, resulting in the construct, Tol2-pATP1A3:GFP (Fig. 9). Finally, the Tol2-pATP1A3:GFP plasmid was sequenced in both directions to ensure correct insertion of the *ATP1A3* promoter. Plasmid DNA for microinjection was purified from culture of transformed DH5α cells (Invitrogen) using the QIAprep Spin Miniprep Kit (Qiagen).

### Preparation of Tol2 transposase mRNA

The pCS-zT2TP plasmid [69], [70] kindly provided by Koichi Kawakami, National Institute of Genetics, Japan, was linearized with *Not*I, gel purified, and used as template for *in vitro* transcription of the Tol2 transposase mRNA. Capped mRNA for microinjection was synthesized using the mMessage mMachine SP6 Kit (Ambion, Inc). The RNA synthesis reaction was treated with TURBO DNase (Ambion) followed by purification using the RNeasy MinElute Cleanup Kit (Qiagen). RNA quality was assessed by denaturing RNA gel electrophoresis using the FlashGel System (Lonza), and RNA content was quantified by spectroscopy.

### Micro-injection and GFP expression

Micro-injection volumes were measured and calibrated by performing 10 injections into a 0.5 µL microcapillary tube (Drummond Microcaps), measuring the amount of liquid using a ruler, and calculating the volume per injection. Five nL of injection mixture containing 50 pg Tol2 transposase-encoding mRNA, and 100 pg Tol2-ATP1A3p:GFP plasmid was microinjected into the centre of the yolk of zebrafish zygotes.

For microscopy, live embryos were sedated with 150 ng/mL tricaine (Aldrich) in E3 and mounted in 1.5% hydroxypropyl methyl cellulose M_n_ 86000 (Sigma-Aldrich). GFP expression in zebrafish embryos and larvae was documented using a Zeiss AXIO Observer.D1 microscope equipped with Zeiss Colibri Illumination System and Zeiss AxioCam MRm.

## Supporting Information

Scheme S1 **Reaction cycle of the Na^+^,K^+^-ATPase.** E1 and E2 are the main conformational states of the enzyme. P indicates phosphorylation. Occluded ions are shown in brackets. Cytoplasmic and extracellular ions are indicated by c and e, respectively.
(TIF)

Table S1 **Oligonnucelotide primer and probes used for cloning and characterization of porcine Na^+^/K^+^-ATPase isoforms α1, α2 and α3.**
(DOCX)
