# Supplementary material for: Molecular Cloning and Characterization of Porcine Na+/K+-ATPase Isoforms α1, α2, α3 and the ATP1A3 Promoter
Source: PLoS One. 2013 Nov 13;8(11):e79127. doi: 10.1371/journal.pone.0079127 (PMC3827302; doi:10.1371/journal.pone.0079127)
Supplement: Table S1 — Oligonnucelotide primer and probes used for cloning and characterization of porcine Na+/K+-ATPase isoforms α1, α2 and α3. (DOCX) [file pone.0079127.s002.docx]

**Table S1**

**Oligonucleotide primer and probes used for cloning and characterization of porcine**

**Na^+^/K^+^-ATPase isoforms α1, α2 and α3**

| **Primer** | **Sequence (5´- 3´)** |
| --- | --- |
| ATP1A1-F | CCGCCACTATGGGGAAG |
| ATP1A1-R | GGAGGGGGTCTAGTAGTAGG |
| ATP1A2-F | CTGCCCCAAGATGGGCCGTG |
| ATP1A2-R | CCAATGGGCTCAGTAGTATGTCTCC |
| ATP1A3-F | ATGGGNGACAAGAAAGATGACAAGG (N=A/T/C/G) |
| ATP1A3-A3F | GAGCCGCCAAGATGGGGGACAAG |
| ATP1A3-R | GGGGATGAGGTCAGTAGTAGG |
| ATP1A3-R1 | CGATCCACAGCAGGATGGAG |
| A3SP1-R | GTTGTATTTCCGGCAGACCTCT |
| A3SP2-R | CTGACATCTTGTGCTCTGTCATAGCC |
| A3SP3-R | CCTTTTTGAGGTCATCC |
| A1RT-F | GACGTGTCCAAGCAAGCTG |
| A1RT-R2 | ATCAAAGATCAGACGACCTTCC |
| ATP1A1-probe#79 | CCTCCTGG |
| A2RT-F2 | TACCAACTGTGTGGAAGGCACT |
| A2RT-R | CCCCCAGGAACACAGCTAC |
| ATP1A2-probe#42 | CATCCAGC |
| A3RT-F | TCAGGCTAAACTGGGACGAC |
| A3RT-R2 | TTTCCTCTGCTCATATGTCCAC |
| ATP1A3-probe#85 | GACCTGGA |
| GAPDH-F | GACTCATGACCACGGTCCATG |
| GAPDH-R | GTCAGATCCACAACCGACACG |
| GAPDH-probe | VIC-CATCACTGCCACCCAGA |
| b_ACT-F | CGTGAGAAGATGACCCAGATC |
| b_ACT-R | TCCATCACGATGCCAGTG |
| b_ACT-probe#56 | TGCTGTCC |
| PA3-F11 | CGCTCTCTGGTGGCACAC |
| PA3-EX1R1 | CCCATCTTGGCGGCTCC |
| HBA1-F | GCTTAACACCTGCTCGAGCT |
| HBA1-R | GAGGTTCCTCTTCTGTAGCAGCT |
| HBA2-F | GCCTCACCAACCAGAGGG |
| HBA2-R | GGATGGTTCATCCTCCATGG |
| HBA3-F | GTCTGACCCACAGCAAAGC |
| HBA3-R | GTCGCCAGAGGGATCGTC |
| XhoI_A3pro-F | CCTCGAGCGCTCTCTGGTGGCAC |
| HindIII_A3pro-R | CCAAGCTTCTTGGCGGCTCCAGAG |
